# Static and Dynamic Mechanical Behaviour of Hybrid-PBF-LB/M-Built and Hot Isostatic Pressed Lattice Structures

**DOI:** 10.3390/ma16093556

**Published:** 2023-05-06

**Authors:** David Sommer, Cemal Esen, Ralf Hellmann

**Affiliations:** 1 Applied Laser and Photonics Group, University of Applied Sciences, Würzburger Straße 45, 63743 Aschaffenburg, Germany; 2 Applied Laser Technologies, Ruhr University Bochum, Universitätsstraße 150, 44801 Bochum, Germany

**Keywords:** hybrid additive manufacturing, lattice structures, hot isostatic pressing, fatigue behaviour

## Abstract

We report on a comprehensive study of the mechanical properties of maraging steel body-centred cubic lattice structures fabricated by a hybrid additive manufacturing technology that combines laser powder bed fusion with in situ high-speed milling. As the mechanical properties of additive manufactured components are inferior to, e.g., cast components, surface modifications can improve the mechanical behaviour. Different hybrid additive manufacturing technologies have been designed using additive and subtractive processes, improving process quality. Following this, mechanical testing is performed with respect to static tensile properties and dynamic stress, hardness, and porosity, comparing specimens manufactured by laser powder bed fusion only to those manufactured by the hybrid approach. In addition, the influence of different heat-treatment techniques on the mechanical behaviour of the lattice structures is investigated, namely solution and aging treatment as well as hot isostatic pressing. Thus, the influence of the superior surface quality due to the hybrid approach is evaluated, leading to, e.g., an offset of about 14–16% for the static testing of HIP lattice structures. Furthermore, the dynamic load behaviour can be improved with a finished surface, heading to a shift of the different zones of fatigue behaviour in the testing of hybrid-built specimens.

## 1. Introduction

The fundamental freedom of design offered by different additive manufacturing (AM) technologies has stimulated a variety of lightweight structures in mechanical engineering over recent years. These lightweight components are often realized by lattice structures in different forms [1,2,3,4]. For instance, periodic structures consisting of unit cells [5,6,7] or gyroid gradients [8,9,10,11] have been utilized for eliminating or relocating material, thus saving weight while maintaining mechanical load capacity [12,13]. Sandwich or infill structures are used for load-dependent design of components for the aerospace industry, modulated for different tensile or compression conditions [14,15]. Based on both experimental studies and simulations, the maximum mechanical load has been correlated to the relative density of different lattice structures [11,16,17].

For metal AM, such as laser powder bed fusion of metals (PBF-LB/M), also referred to as selective laser melting (SLM), the achievable surface accuracy and roughness of components are, in general, inferior to conventional manufacturing methods, limiting applications with demanding requirements in terms of shape and surface conditions. In addition, surface defects and roughness influence mechanical properties such as, e.g., the tensile and fatigue strength. Hence, subtractive surface post-processing is essential for PBF-LB/M components to condition shape and surface properties. This, however, might be challenging for lattice structures due to inaccessible, inner lying areas within the complex lattice arrangement.

In this respect, Zheng et al. and Sarkar et al. investigated the mechanical properties of PBF-LB/M-built components and demonstrated that these are, generally, inferior to conventionally fabricated components [18,19], while surface modifications can improve properties such as, e.g., the fatigue life [20,21,22,23].

To circumvent these disadvantages of PBF-LB/M, various hybrid additive manufacturing processes, generally consisting of an additive and a subtractive process, have been developed [24,25,26]. A very promising hybrid approach is the combination of regular PBF-LB/M as an additive process with high-speed-milling as a subtractive process, capitalizing the advantages of both technologies while evading the disadvantages of the PBF-LB/M-process. In particular, in situ high-speed milling integrated into the powder bed process fosters the capabilities of laser powder bed fusion towards superior surface quality and geometrical accuracy [27,28,29] with the unique advantage of providing access to component surfaces during the 3D-building phase which might be inaccessible after the component is finished.

In addition, with respect to the mechanical properties of metal structures built by PBF-LB/M, the effects of a post process heat treatment on the microstructure and porosity, hardness, yield and fatigue strength have specifically been addressed [20,30,31,32,33,34,35,36,37,38]. It has been revealed that a post PBF-LB/M heat treatment increases the mechanical load capacity [39,40,41]. In particular, solution and aging treatment [42,43] or hot isostatic pressing [44,45] have been proven to reduce porosity and positively modify the microstructure of PBF-LB/M-built parts, in turn leading to improved mechanical properties.

Against this background of optimizing PBF-LB/M of lattice structures for lightweight applications and applying heat treatments to metal AM components, the mechanical properties of body-centred cubic lattice structures are studied. Focussing on the maximum applicable static load and fatigue behaviour upon dynamic load, sole PBF-LB/M-structures are compared with a hybrid process, comprising PBF-LB/M and in-situ 3-axis micro milling. In addition, the effect of different heat-treatments, namely a solution and aging treatment as well as hot isostatic pressing, are investigated by studying porosity, hardness, and microstructure.

## 2. Materials and Methods

### 2.1. Hybrid-PBF-LB/M Milling System

**Figure 1:**
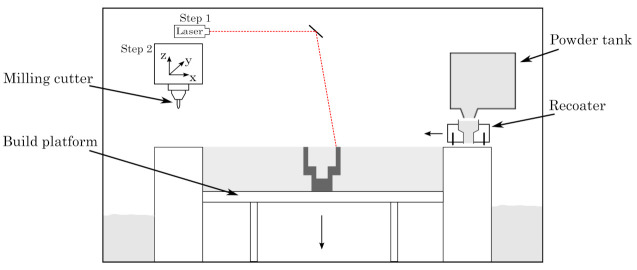
Schematic illustration of the hybrid additive manufacturing unit.

**Figure 2:**
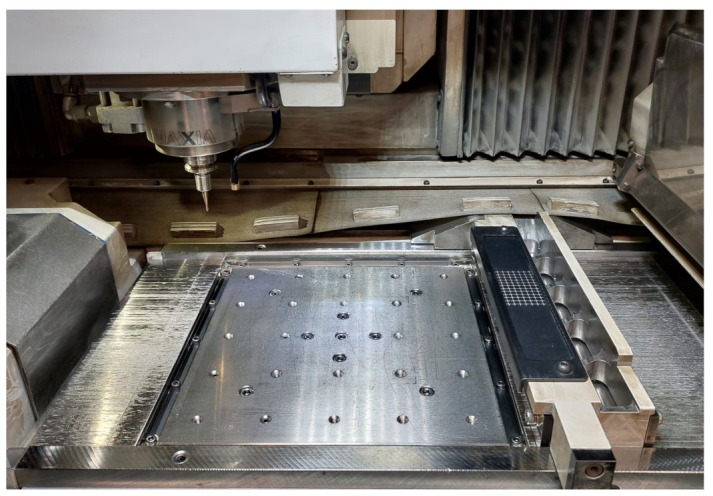
Hybrid additive manufacturing unit, showing the build plate with the recoater and the milling spindle.

Body-centred cubic lattice structures (cf. Section 2.2) are fabricated by additive and hybrid additive manufacturing using a Lumex Avance-25 (Matsuura Machinery GmbH, Wiesbaden, Germany). The machine combines conventional laser powder bed fusion with in situ three-axis high-speed milling, as schematically illustrated in Figure 1 and depicted in Figure 2.

For the milling process, the conventional PBF-LB/M process sequence is paused after several layers (typically ten), allowing access of the milling cutter to all contours of the specimen built thus far. This in turn allows the generation of finished components with superior surface roughness, including the machining of undercuts or internal structures.

The hybrid manufacturing process is conducted under nitrogen atmosphere with less than 3% oxygen level to avoid oxidation, with the build platform being kept at 50 °C to prevent deformation by curling due to thermal stress. The maximum build volume is 250 × 250 × 185 mm${}^{3}$ in width, depth, and height.

Since the PBF-LB/M-process does not fundamentally differ from industrial standard and this comparative study focuses on the tensile fatigue stress behaviour of the PBF-LB/M- and hybrid-built components, previously optimized and reported process parameters for this machine setup are applied for the manufacturing of the specimens (cf. Table 1) [29]. The machine is equipped with an Ytterbium fibre laser (SPI Lasers plc, Southampton, UK) operating at a wavelength of 1070 µm with a focal diameter of 200 µm.

For high-speed milling, the integrated spindle operates at up to 45,000 revolutions per minute and a maximum torque of 1.31 Nm. In addition, a twentyfold tool magazine is available for the change of the milling device during operation. The milling cutters used in this study are solid carbide cutting tools with a nano coating, consisting of aluminium, titanium, and silicone for the reduction of tool wear (Mitsubishi Materials Corporation GmbH, Meerbusch, Germany). The wear characteristics reported in [46] are reinforced by virtue of the dry milling process. Due to the in situ application of the milling process inside the powder bed, no cooling lubricant can be used, leading to elevated temperature conditions and increasing tool wear [47,48,49]. To analyse the application of the lattice structures, previously determined milling parameters are used, given by Table 2 [29].

**Figure 3:**
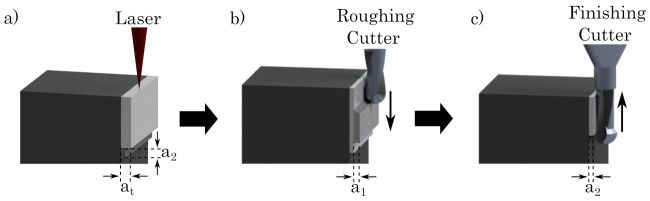
Two stage milling process with two different milling parts for the hybrid system. (**a**) PBF-LB/M process; (**b**) Roughing process; (**c**) Finishing process.

As reported before [27,46], the high-speed milling process starts after several built layers, interrupting the PBF-LB/M-process. In our study ten layers with a height of 50 µm each are built before starting the milling process. Within the PBF-LB/M-process, a material allowance of $a_{t}$ = 150 µm is added to the component structure, being removed gradually by the usage of two different milling cutters, realizing a roughing and a finishing sequence (cf. Figure 3b,c). At first, $a_{1}$ = 120 µm of the material allowance are detached by the roughing cutter, machining the surfaces from the last built layers downwards. Afterwards, the finishing cutter removes the remaining $a_{2}$ = 30 µm of the material allowance, improving the surface roughness and the final geometrical accuracy.

Contrary to the roughing cutter, the finishing cutter is working from the bottom to the top of the component as well as the last built layers are spared for the next process cycle. (cf. Figure 3a, where the finishing cutter has left several layers of $a_{2}$). This geometrical shift of the milling processes is chosen to avoid thermal distortion as well as to optimize geometrical accuracy and superior surface quality. Due to the PBF-LB/M-process, a heat input is generated, evolving a thermal gradient within the built part. The upper layers exhibit a higher temperature than the layers below, which have already cooled down [50,51]. By virtue of the thermal conditions, a start at the bottom layers working upward is advised and the last built layers should be omitted [52,53,54].

### 2.2. Specimen Design and Material

**Figure 4:**
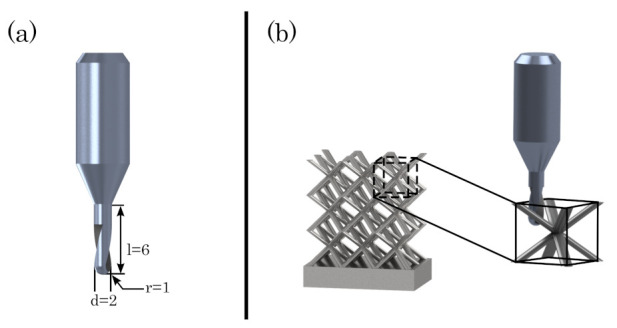
(**a**) Dimensions of the milling cutter, (**b**) milling of lattice structures.

To study the effects of an improved surface quality provided by the hybrid process, a body-centred cubic unit cell has been chosen for the lattice structures, as it allows access of the milling cutter and as it represents an often chosen and well-studied structure. The dimensions of the unit cell are 5 × 5 × 5 mm, and the specimens are designed with 3 × 3 × 2 (width × height × depth) cells and a strut diameter of 1 mm, leading to a test specimen with about 13% relative density (cf. Figure 4b). Within the hybrid approach, the manufacturability of the specimens is determined by the three-axis milling system and the diameter of the used milling cutter. In this study, the used milling cutter has a diameter of 2 mm, exhibiting the geometry, shown in Figure 4a). Combined with the three-axis milling system, the milling cutter is machining the specimens on the top and besides (cf. Figure 4b), the undercut surfaces cannot be milled by virtue of the dimensions of the unit cell.

The study is performed processing maraging tool steel X3NiCoMoTi18-9-5 (1.2709, Matsuura Machinery GmbH, Wiesbaden, Germany), a high-nickel steel exhibiting a distinctive mixture of high strength and high hardness, combined with ease of welding. It is preferably employed in the die and tooling industry as well as in structural and aerospace applications [55,56]. The use of maraging steel in PBF-LB/M is also stimulated by the potential of generating cooling channels for the reduction of cycle times in injection moulding [57] of plastics and high-pressure casting of metals [58].

### 2.3. Post-Processing Procedures

As thermal post-processing of maraging steel, in general, improves mechanical properties of the built parts, the influence of solution treatment combined with aging treatment (SAT) as well as the impact of the hot isostatic pressing (HIP) are studied. For the SAT, a Nabertherm LH 120/12 batch furnace (Nabertherm, Lilienthal, Germany) is employed, enabling heat treatments up to 1200 °C with a maximum heat rate of 10 °C/min. The specimens, completely processed without process gas, are heated to 940 °C at the maximum heat rate, held for 30 min and cooled down to approximately 25 °C with a controlled cooling rate of 10 °C/min (solution treatment). Subsequently, for the aging treatment, the furnace is heated up to 550 °C, held for 4 h and followed by a natural cooling.

The HIP-process is conducted with a Quintus QIH (Quintus Technologies AB, Västeras, Sweden) with a maximum furnace temperature of 1400 °C and a maximum process pressure of 200 MPa. Argon gas is utilized as a process gas. The process starts with a heating up to 1035 °C with a heating rate of 15 °C/min, increasing the pressure to 150 MPa at the same time. This step is followed by a controlled cooling with 10 °C/min down to 940 °C, whereby the previously explained SAT procedure is finished.

**Figure 5:**
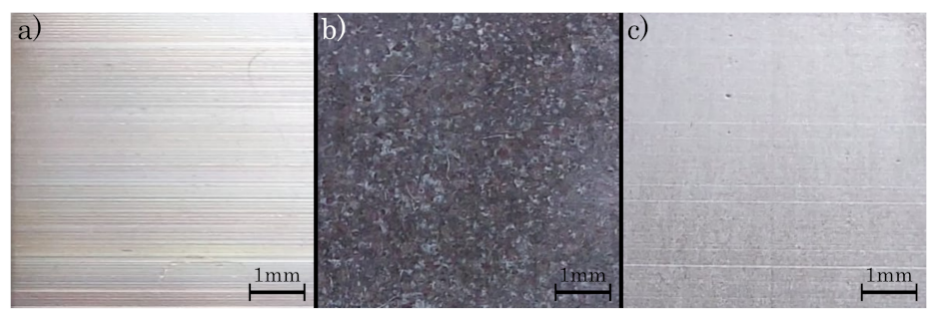
Surfaces of milled specimens: (**a**) as-built, (**b**) SAT and (**c**) HIP.

The surface properties change within the different heat-treatment procedures (cf. Figure 5). For the SAT, the surface of the specimens shows an effect of blushing, while the HIP specimens obtain a dull appearance. In addition, at the as-built specimens as well as at the HIP specimens, the milling paths are visible, showing a frequent waviness.

### 2.4. Sample Characterization

The mechanical behaviour of the built specimens is tested in the dependence of uniaxial static and dynamic load. For the compression tests with static load, an AG-X plus universal testing machine (Shimadzu, Kyoto, Japan) is used, applying a maximum force of 50 kN due to a precision ball-screw drive. For every state of the heat treatment, machined and PBF-LB/M-built, a batch of three specimens is tested, increasing the statistical significance.

The dynamic testing is executed with a StepLab UD020 (Step Engineering, Resana, Italy) with a maximum compression of 14 kN with a sinusoidal stress-time sequence at a maximum fatigue test frequency of 100 Hz. Analogous to the static tests, three specimens are tested for every level of amplitude, recording the number of cycles until failure.

For the characterization of the microstructure of polished and etched surfaces, a laser scanning microscope VX-2000 (Keyence, Osaka, Japan) is employed. For the examination of the microstructure, the specimens are acid treated with an etching agent according to Adler [59]. Fractographical analysis of cracks appearing during the mechanical testing is conducted with a scanning electron microscope (Maia-3, TESCAN, Dortmund, Germany), using the secondary electron detector (SE) and a SEM voltage of 3 kV.

## 3. Results and Discussion

In the following, the results of the comparative study between PBF-LB/M-built and hybrid-built lattice structures are shown and discussed. Firstly, mechanical properties with respect to static and the dynamic load for the as built structures are discussed. Secondly, the influence of SAT and HIP is evaluated for both PBF-LB/M-built and hybrid-built lattice structures.

### 3.1. Static Testing

**Figure 6:**
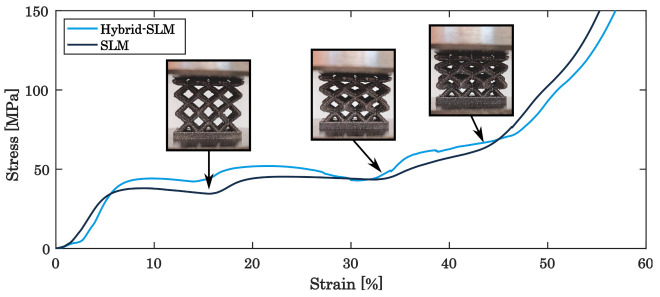
Stress–strain curve of the as-built lattice structures.

Upon static load testing, qualitatively the typical performance for maraging steel is observed, revealing a ductile behaviour [60]. In addition, the consecutive collapse of the three individual layers of the built lattice structure is clearly seen in the stress–strain-diagram, until the entire specimen is densely compressed in test direction without entire rupture of the struts, in turn increasing the stress to the maximum applied load, as depicted in Figure 6 [23,33,54].

**Figure 7:**
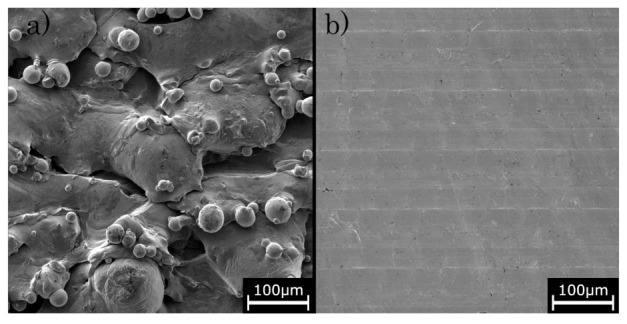
SEM images of (**a**) PBF-LB/M-built surface and (**b**) high-speed milled surface.

Comparing the sole PBF-LB/M-built and hybrid-built lattice structures, Figure 5 also shows that the in situ milled lattice structure withstands higher loads, consistently about 14–16% higher, before breaking the single layers than the PBF-LB/M-built structures. The strain and fracture behaviour of PBF-LB/M-built components is significantly influenced by the quantity and dimensions of surface defects [61,62,63]. Subsequently, the specific energy absorption (SEA) of the hybrid additive manufactured specimens is increased, scaling the applied load to the mass of the specimens [64,65,66]. While sole PBF-LB/M-built structures show a SEA of about 67.1 kJ/kg, the energy absorption can be raised to about 72.1 kJ/kg due to the surface finish. As shown in the SEM images in Figure 7, the PBF-LB/M-built surface reveals distinct irregularities and defects, associated by adhering powder particles, insufficiently molten regions and superficial cracks, which in turn weaken the mechanical resistance of the specimen. Contrary to this, the in situ machined parts do not show such large defects or cracks on the surface. While the surface roughness, in general, is superior to the PBF-LB/M-built surface roughness, the z-pitch of the milling process can be observed as an irregularity (Figure 7b). The surface quality can be improved to an average surface roughness of $R_{a}$ = 1.5 –2 µm, as reported before [46], getting assigned to lead to the superior load capacity of the hybrid-built structures.

**Figure 8:**
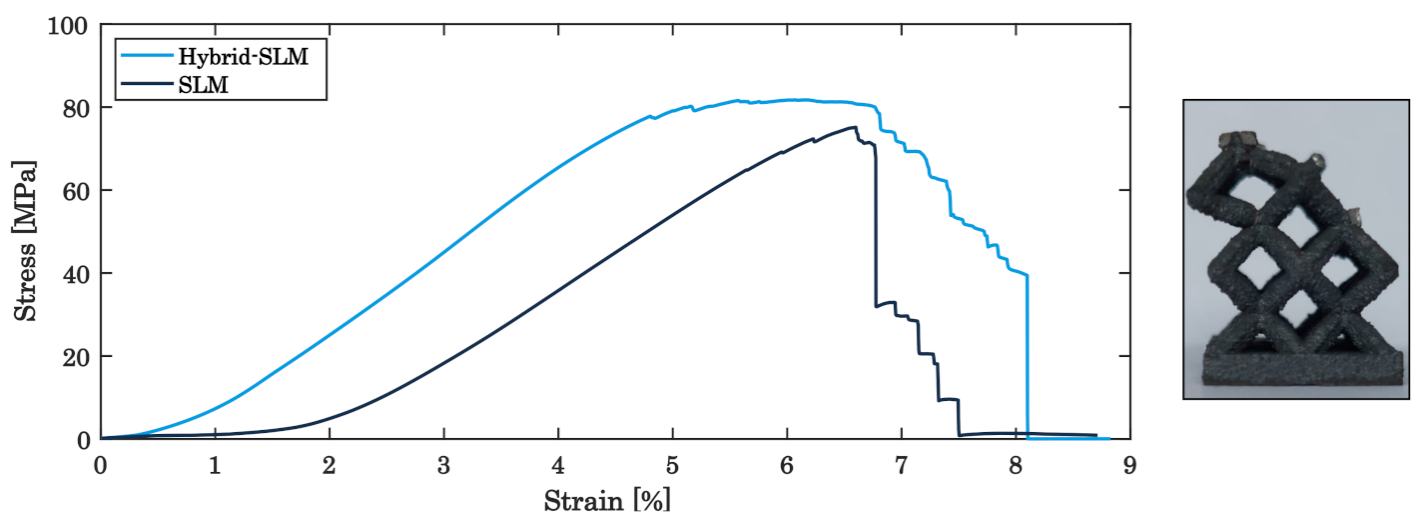
Stress–strain diagram for the comparison of Hybrid PBF-LB/M- and PBF-LB/M-built specimens for SAT lattice structures.

After heat treatment with the SAT process, the specimens reveal a distinctively different mechanical behaviour during static testing. As shown in Figure 8, instead of the layer-wise collapse of the different unit cells, the specimens fail completely at a maximum load of 75 MPa (unmachined) and at 81 MPa (machined), exhibiting typical shear fractures [54]. This difference in the mechanical behaviour can be attributed to a change in microstructure as it is expected after the SAT. The martensite (α) phase is dominant for the PBF-LB/M as-built state of maraging steel, indicating only a fractional amount of the austenite (γ) phase [67]. During solution treatment, the γ phase completely merges into the α phase, converting back within aging. The ductile behaviour, observed for the as-built specimen, is significantly reduced and the performance during static load tests is dominated by the austenite phase, leading to a very strong but brittle material [68]. In addition, the hybrid-processed specimens reveal a superior stress–strain response as compared to the sole PBF-LB/M-built parts, attesting the effect of an improved surface condition on the strain and fracture behaviour.

**Figure 9:**
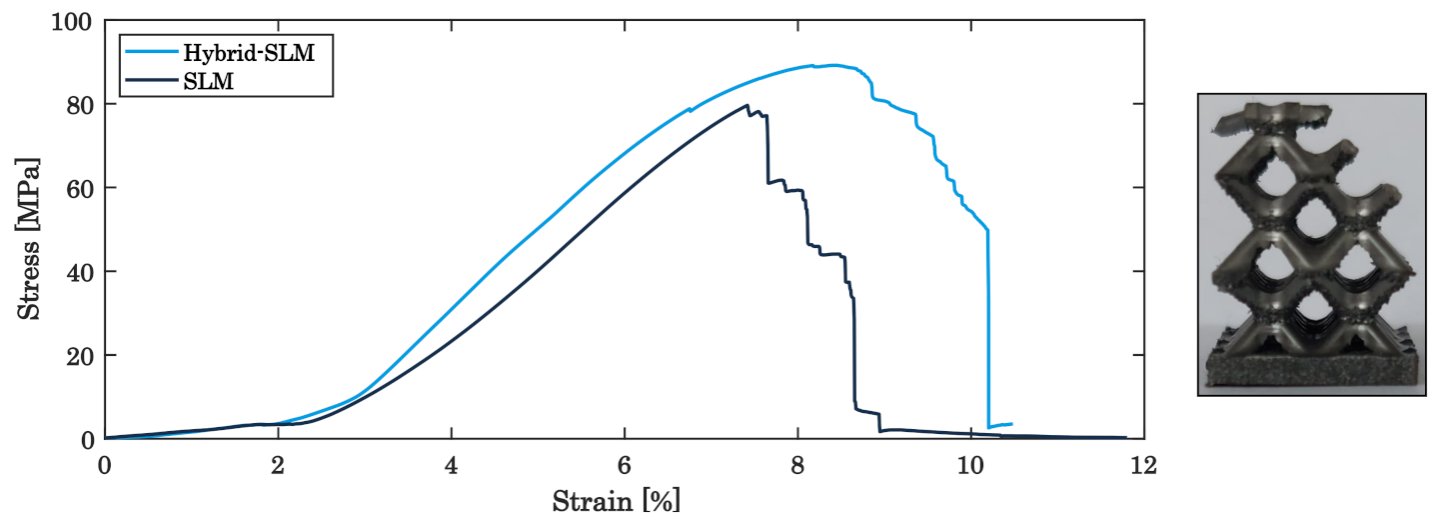
Stress–strain diagram for the comparison of Hybrid PBF-LB/M- and PBF-LB/M-built specimens for HIP lattice structures.

Qualitatively, a similar behaviour is found after HIP of the lattice structures, which fail at a maximum load of 79 MPa (sole PBF-LB/M-built) and 90 MPa (in situ milled), minimizing the ductile material structure as well as the SAT specimens (c.f. Figure 9). The increase in the maximum load of the hybrid-built specimens of about 12% shows an improvement, which is expected to arise from the superior surface quality [69]. In comparison to the SAT specimens, the maximum load for the failure has been increased with about 10 MPa, as reported by previous studies similarly for the comparison of SAT and HIP specimens [70]. The increase of the maximum load can be attributed to the improved material properties due to the HIP process, quantified by the porosity and the hardness of the tested material, measured additionally at cubic test specimens.

**Figure 10:**
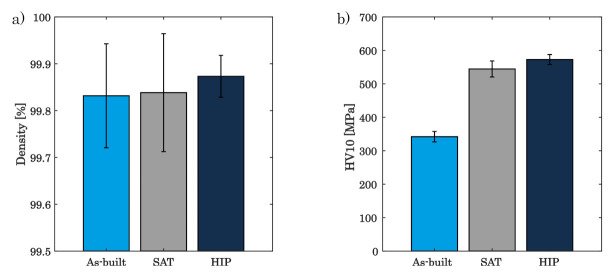
Comparing the as-built, SAT and HIP states for (**a**) density and (**b**) hardness.

As shown in Figure 10a, the density of post processed components can be raised even though the porosity of as-built components is already high, being about 99.8%. Yet, the density of the SAT-processed specimen reaches about 99.84%, increasing marginally to almost 99.9% in succession of the HIP process. A more pronounced advancement in material properties, however, is achieved for the hardness (c.f. Figure 10b). While the hardness for the PBF-LB/M-built components is approximately at 345 MPa, post-processing improves this characteristic to about 550 MPa for the SAT specimen and to 570 MPa for the HIP specimen.

**Figure 11:**
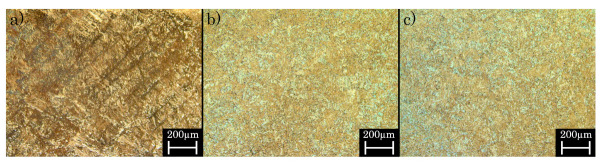
Microstructure of the (**a**) as-built, (**b**) SAT and (**c**) HIP specimens.

While these experimental results indicate an improvement of the mechanical behaviour induced by the heat treatment, they simultaneously lead to a modification of the microstructure of the material [71]. The microstructure of the as-built specimens is unidirectional and dominated by a very fine-grain structure (Figure 11). Due to the different heat treatments, the microstructure changes, leading to a more coarse-grained structure and a dissipated alignment for both heat treatments [60].

### 3.2. Dynamic Testing

Dynamic mechanical testing is performed according to Section 2.4 with a maximum load of 14 kN at a frequency of 50 Hz. For every level of amplitude, three specimens are tested until the final failure occurs, the average being depicted in a Wöhler diagram.

**Figure 12:**
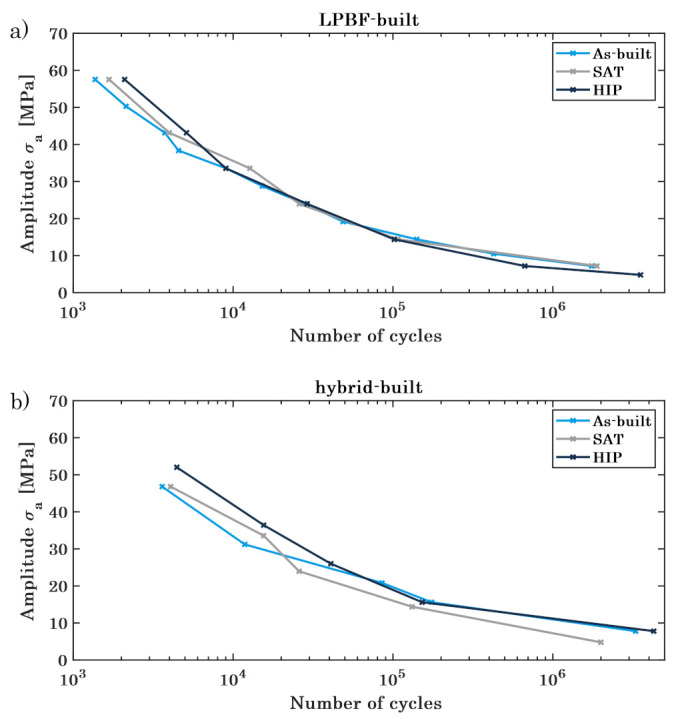
Wöhler diagram for the (**a**) PBF-LB/M-built and (**b**) hybrid-built specimens, each comparing the as-built, SAT and HIP states.

Figure 10 visualises the resulting Wöhler diagrams for the PBF-LB/M-built (Figure 12a) and the hybrid built (Figure 12b) lattice structures, comparing the specimens in the as-built state with the different heat treatment processes. The fatigue behaviour changes in dependence on the cycle number for the various batches of specimens, determining the areas of strength. For both, the PBF-LB/M-built and the hybrid-built lattice structures, the SAT- and HIP-treated specimen reveal a better performance in the low cycle fatigue (LCF) regime, decreasing to assimilable cycle numbers in the section of the high cycle fatigue (HCF). The heat-treated specimens prove a comparable performance for the very high cycle fatigue (VHCF), whereas the as-built structures demonstrate an inferior performance. As for the static load behaviour, the improved hardness and porosity of the heat-treated specimens, especially the HIP specimens, is advantageous for the LCF. With higher cycle numbers, the material performance of the as-built lattice structures is more ductile, raising the VHCF.

**Figure 13:**
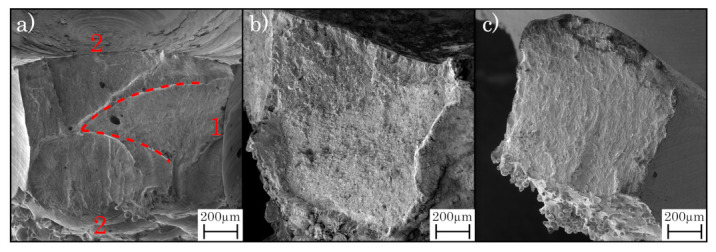
Crack analysis for (**a**) as-built (zone 1: endurance failure, zone 2: forced fracture, getting divided by dashed line), (**b**) SAT and (**c**) HIP specimens for the same applied load.

For the as-built specimens, the machined structures show a similar overall trend of fatigue behaviour, yet exhibiting an offset with higher cycle numbers for all different sections of strength. This difference can be attributed to the superior surface quality of the machined specimens, as stated before. Figure 13a depicts the crack-analysis for the as-built lattice structures, revealing an endurance failure (zone 1) as a beginning of the crack, including an internal void, possibly enabling the endurance failure. Further, a forced fracture (zone 2) is depicted, causing complete fracture of the specimen. Due to the ductile material structure of the maraging steel in the as-built state, an endurance failure can develop, weakening the structure with every cycle until its complete failure [72].

**Figure 14:**
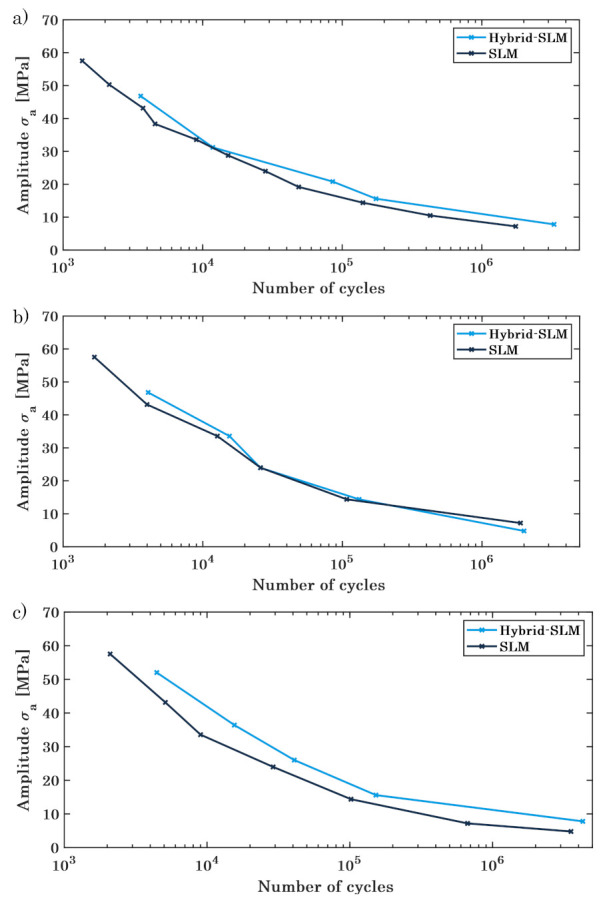
Wöhler diagram for the (**a**) As-built, (**b**) SAT and (**c**) HIP specimens, comparing machined and unmachined specimens.

The SAT structures exhibit almost identical numbers of cycles for the different loads, differing at the beginning of the low cycle fatigue but converging at the following high cycle fatigue, as shown in Figure 14b. Contrary to the as-built specimens, the fracture plain shows a big part of a forced fracture and excludes the endurance failure. The fracture surface only reveals an increased roughness without showing a structure or a development of the fracture (cf. Figure 14b). Due to the heat treatment, the material hardness increased, yet it became more fracturable, leading to a complete failure at a load that excels the maximum capacity.

Similar to the SAT specimens, the HIP lattice structures demonstrate a big part of a forced fracture without any indication of a cyclic developing weakness (cf. Figure 13c). The endurance failure is, for small and high loads, not identifiable at the fracture plain, showing only a little number of porosities, subsequently increasing the maximum load for the lattice structures. Furthermore, the hybrid-built specimens excel the PBF-LB/M-built in the number of the cycles, retaining the offset during all sections of the fatigue testing. The load for the low cycle fatigue is about 10 MPa higher for the machined specimens as for the PBF-LB/M-built as well as the number of cycles in the high cycle fatigue differs about 25,000 at the same applied load (Figure 14).

## 4. Conclusions

The static and dynamic mechanical behaviour of PBF-LB/M- and hybrid-PBF-LB/M-built lattice structures of maraging tool steel, with the hybrid approach consisting of standard PBF-LB/M combined with in situ high-speed milling, was studied. In addition, the effect of different heat-treatment processes, namely solution treatment combined with an aging treatment process as well as hot isostatic pressing, have been examined, comparing the PBF-LB/M-built and machined specimens. BCC unit cells with a relative density of about 13% were chosen as lattice structures to evaluate the particular influence of the superior surface quality of the in situ milled lattices. For the evaluation of the mechanical load behaviour, static testing has been performed, showing a typical ductile load behaviour for the as-built specimens, while the heat-treated specimens perform with a strong but brittle behaviour, developed due to the post processing.

However, the difference between the PBF-LB/M-built and the hybrid-built specimens persists whether the components are post processes or in the as-built state. As a result of the subsequent machining, the maximum load of the milled specimens exceeds that those generated solely with the PBF-LB/M. The in situ milling can eliminate surface defects and superficial cracks, increasing the mechanical behaviour within the static testing. Further, the machined specimens exceed the PBF-LB/M-built at dynamic testing, as an offset of cycle-numbers is persisting nearly continuously over the different sections of fatigue testing. Partially, milled specimens can be assigned to the VHCF, while the PBF-LB/M-built components fall into the range of HCF at the same load. Overall, this study outlines a comprehensive evaluation of the mechanical properties of hybrid-built lattice structures for static and dynamic testing, highlighting the superior load behaviour for the hybrid approach, combining Laser powder bed fusion and in situ high-speed milling as an innovative manufacturing technology.

## Figures and Tables

**Table 1:** Laser powder bed fusion parameters.

	Laser Power [W]	Scan Speed [mm/min]	Hatch Distance [µm]
**Area**	320	700	0.12
**Contour**	320	1400	-
**Support**	320	700	0.12

**Table 2:** Milling process parameters.

	Z-Pitch [µm]	Spindle Speed [rot/min]	Feed Rate [mm/min]
**Roughing Cutter**	0.15	30,000	2000
**Finishing Cutter**	0.1	30,000	1600

## Data Availability

Not applicable.
